# The Effect of Psychotropic Agents on Sick Sinus Syndrome: A Case Report

**DOI:** 10.7759/cureus.113954

**Published:** 2026-08-04

**Authors:** Lynn Fadel, Ansar Malik, Sudhakar Pemminati

**Affiliations:** 1 Biomedical Education, California Health Sciences University College of Osteopathic Medicine, Clovis, USA; 2 Specialty Medicine, Thriving Minds Psychiatric Services of California, Covina, USA; 3 Pharmacology, California Health Sciences University College of Osteopathic Medicine, Clovis, USA

**Keywords:** buspirone, cyp2d6 inhibition, flecainide, mirtazepine, qt prolongation, quetiapine, sick sinus syndrome

## Abstract

Sick sinus syndrome (SSS) presents significant challenges in psychiatric medication management because of the potential for worsening cardiac conduction and interactions with antiarrhythmic therapy. A 35-year-old woman with generalized anxiety disorder (GAD) and insomnia was treated with bupropion, hydroxyzine, melatonin, and intermittent quetiapine, alongside comorbid SSS and supraventricular tachycardia managed post-ablation with metoprolol. A review of her regimen identified several cardiac safety concerns: quetiapine’s potential interference with flecainide, hydroxyzine-associated QT prolongation, and bupropion’s CYP2D6 inhibition that could increase flecainide levels. Management prioritized minimizing arrhythmogenic risk by discontinuing quetiapine, restricting hydroxyzine to low doses, and continuing melatonin, supplemented by stress testing and reassessment of psychotropic options. This case highlights the importance of careful psychotropic selection in patients with SSS, favoring lower-risk agents, such as mirtazapine, which is one of the safest among several possible alternatives, while maintaining close ECG monitoring and coordinated cardiology and psychiatry collaboration to reduce drug-drug interactions and optimize safety.

## Introduction

Sick sinus syndrome (SSS), or sinus node dysfunction, encompasses a spectrum of arrhythmias caused by impaired impulse generation or conduction at the sinoatrial node. It is most common in older adults and may manifest as sinus bradycardia, sinus pauses, sinoatrial exit block, or alternating bradycardia and tachycardia (tachy‑brady syndrome) [1]. Symptoms typically reflect cerebral hypoperfusion, such as syncope, presyncope, and lightheadedness, but may also include palpitations, fatigue, and exercise intolerance [1]. Diagnosis requires correlating symptoms with documented bradyarrhythmias while excluding reversible extrinsic contributors, including medications and metabolic disturbances. For symptomatic SSS, permanent pacemaker implantation remains the first‑line therapy, accompanied by removal of reversible factors when feasible [1].

Supraventricular tachycardia (SVT) comprises rapid arrhythmias originating above the His bundle, including atrioventricular (AV) nodal reentrant tachycardia, AV reentrant tachycardia, and atrial tachycardia [2]. Patients commonly present with palpitations, chest discomfort, anxiety, dyspnea, or fatigue. Diagnosis is confirmed through ECG or ambulatory monitoring. Acute management includes vagal maneuvers, adenosine, and rate‑controlling agents, while catheter ablation is the preferred strategy for recurrent or symptomatic SVT [3].

Anxiety and insomnia are highly prevalent in patients with cardiovascular disease, including those with SSS and SVT. These psychiatric comorbidities can exacerbate cardiac symptoms and complicate management. First-line treatments for anxiety disorders include selective serotonin reuptake inhibitors (SSRIs) and selective norepinephrine reuptake inhibitors (SNRIs), with sertraline favored for its lower risk of QTc prolongation. Tricyclic antidepressants and monoamine oxidase inhibitors (MAOIs) should be avoided due to their arrhythmogenic potential. For insomnia, cognitive behavioral therapy for insomnia (CBT-I) is recommended as first-line therapy, with pharmacologic options such as dual orexin receptor antagonists (daridorexant, lemborexant, suvorexant) considered safer than benzodiazepines, zolpidem, zaleplon, or sedating antidepressants, which carry significant cardiac and cognitive risks [4,5].

SSS presents unique challenges for psychiatric drug management, as many psychotropic agents can worsen conduction abnormalities or interact with antiarrhythmic therapies. Medications such as lithium, tricyclic antidepressants, and certain antipsychotics (e.g., risperidone) are known to exacerbate bradycardia and conduction delays and should be avoided in SSS [6]. Additionally, negative chronotropic drugs (beta blockers, calcium channel blockers, digoxin, and antiarrhythmics) commonly used for SVT may further depress sinus node function [3]. The risk of drug-induced arrhythmia is heightened in patients with underlying conduction disease, necessitating careful selection and monitoring of psychiatric medications.

Coordinated care between cardiology and psychiatry is essential to optimize safety and address mental health needs in patients with SSS and SVT. Multidisciplinary collaboration ensures that psychiatric symptoms are managed without compromising cardiac function and that medication regimens are tailored to minimize arrhythmia risk. Shared decision-making, regular ECG monitoring, and ongoing communication between specialties are critical for safe and effective treatment. 

This article was previously presented as a poster at the 2026 Annual Meeting, Central California Psychiatric Society, March 27-29, 2026.

## Case presentation

A 35-year-old woman presented with generalized anxiety disorder (GAD) and chronic insomnia, whose psychiatric management was complicated by underlying SSS with supraventricular tachycardia (SVT). SSS is most commonly prevalent in the elderly population; however, this patient was demonstrating an atypical presentation due to its early onset. Her psychiatric symptoms had been managed with bupropion, hydroxyzine, nightly melatonin, and intermittent quetiapine for sleep. Cardiac history was significant for symptomatic bradyarrhythmia and recurrent SVT episodes, previously treated with catheter ablation and currently stabilized with β-blocker therapy, specifically 75 mg of metoprolol daily. She presented with ongoing fatigue, nocturnal sleep disruption, and elevated stress levels despite medication adherence. Over several weeks, she reported difficulty sustaining rest at night, described as frequent awakenings, and a persistent sense of physiologic overactivation during the day.

During medication review and interdisciplinary discussion, concerns emerged regarding pharmacologic interactions between her psychiatric regimen and antiarrhythmic therapy. Quetiapine was identified as potentially unsuitable due to possible interaction with flecainide, raising the risk of impaired conduction and arrhythmogenic effects. QTc prolongation is a common side effect to note when using quetiapine and flecainide is a class IC antiarrhythmic agent that slows cardiac conduction by targeting the sodium channels on cardiac tissue, therefore prolonging the QTc interval as well [7,8]. Hydroxyzine, though subjectively helpful for anxiety and sleep, was also highlighted for its QT-prolonging potential, particularly in patients with preexisting conduction pathology [9]. This adverse effect can occur at varying doses, with a case study reporting occurrence at 12.5 mg in an 82-year-old woman with complete atrioventricular block, but QTc prolongation typically occurs at doses greater than 100 mg per day [9,10]. Bupropion further complicates management, as its strong CYP2D6 inhibition can increase circulating flecainide levels, thereby heightening the pro-arrhythmic risk in the context of SSS [11]. The patient reported no previous episodes of syncope or palpitations related to psychiatric medication use, but cardiology advised caution given the cumulative interaction burden.

A collaborative management plan was developed to prioritize cardiac safety while preserving psychiatric stability. Melatonin was continued without modification, and hydroxyzine was limited to low-dose, as-needed use with ECG monitoring. Quetiapine was discontinued, and alternative agents were evaluated with a focus on medications with minimal QT liability and reduced CYP2D6 inhibition. Sertraline and mirtazapine may be safer alternatives for long-term anxiety management and sleep regulation, warranting gradual titration and monitoring; larger studies are required to confirm these findings. A treadmill stress test was scheduled to evaluate chronotropic tolerance, and follow-up electrocardiographic surveillance was arranged to assess conduction response to any medication adjustments.

Once the appropriate cardiac workup was completed, the patient was ready to begin trying alternative treatment for her insomnia. Ramelteon, a melatonin receptor agonist, was attempted. However, the patient discontinued it due to the lack of efficacy and had difficulty picking up refills due to cost barriers. Ultimately, with the agreement of her cardiologist, the patient attempted suvorexant, an orexin receptor antagonist, and is currently responding well to it.

## Discussion

This case illustrates the clinical challenge of treating psychiatric illness in individuals with SSS, particularly when antiarrhythmics intersect with psychotropic agents that carry QT-prolonging risk or metabolic interactions. Medications such as hydroxyzine and quetiapine, though commonly used for anxiety and sleep, may introduce significant cardiologic risk when combined with class IC agents. Similarly, bupropion’s inhibition of CYP2D6 introduces a mechanism for unintentional flecainide accumulation, underscoring the importance of metabolic awareness in psychopharmacology [11].

The patient’s clinical course highlights the necessity of integrated cardiology-psychiatry collaboration, proactive ECG monitoring, and individualized medication selection. A broader clinical recognition of arrhythmia-psychiatric medication interaction profiles is needed, and emerging cases may help guide safer prescribing frameworks for patients with conduction system disease. Further studies should explore optimized psychotropic strategies for SSS patient populations, including comparative risk profiling and long-term outcomes associated with low-interaction regimens.

Safer alternatives, such as sertraline or mirtazapine, may be preferable. Continuous monitoring with ECGs and interdisciplinary collaboration is crucial. Sertraline is preferred because it has a low risk of QTc prolongation and arrhythmia, with extensive safety data in patients with cardiovascular disease. It is recommended by multiple guidelines for anxiety and depression in cardiac populations and is less likely to interact with antiarrhythmic therapies or worsen conduction abnormalities. Mirtazapine is also favored for its minimal impact on cardiac conduction, sedative properties useful for insomnia, and generally benign cardiac profile. Although rare cases of heart block have been reported, its overall arrhythmia risk is lower than that of tricyclics or antipsychotics [12]. Direct comparative data on mirtazapine’s arrhythmia risk in SSS/SVT are limited, and further research is warranted. Nonetheless, current evidence supports the preferential use of sertraline and mirtazapine over agents with higher arrhythmogenic potential in patients with cardiac conduction disorders. Though mirtazapine may be safe when taken on its own, it must be prescribed with caution as cardiac concerns may arise when combined with other QTc-prolonging agents in patients with a history of cardiovascular disease [13]. The comparison of the effects of different psychotropic drugs on cardiac concerns is summarized in Table 1 [14-16].

**Table 1:** Comparison of the effects of different psychotropic drugs on cardiac concerns SSRI: Selective serotonin reuptake inhibitors, SVT: supraventricular tachycardia, AV: atrioventricular.

Drug Class	Medication Name	Use with caution/avoid high doses in Sick Sinus Syndrome/Supraventricular Tachycardia	Mechanism of cardiac risk
Antidepressants [14,15]	Tricyclic antidepressants (TCA) (e.g., amitriptyline, nortriptyline)	Yes	Sodium channel blockade: TCAs slow cardiac depolarization and prolong conduction (PR, QRS, QT). This increases the risk of atrioventricular (AV) block, bradyarrhythmias, and reentrant tachyarrhythmias. Anticholinergic effects: These commonly produce sinus tachycardia, which can precipitate or worsen SVT episodes. Inhibition of norepinephrine reuptake: Increases sympathetic tone, further promoting tachyarrhythmias.
	Citalopram, escitalopram [16]	Yes (high doses)	QT prolongation, risk of torsades de pointes. Citalopram is an SSRI, but unlike most SSRIs, it has dose‑dependent QT prolongation. This creates a substrate for arrhythmias that are particularly dangerous in patients with existing conduction disease. QT prolongation: increases risk of torsades de pointes and other ventricular arrhythmias. Bradycardia risk: citalopram can cause dose‑related bradycardia, which worsens SSS. Conduction slowing: can exacerbate sinus node dysfunction and AV nodal delay. Arrhythmia triggering: QT prolongation and bradycardia can precipitate tachy‑brady episodes in SSS and increase susceptibility to SVT.
	Fluoxetine [17]	Interacts with flecainide and inhibits its metabolism	Flecainide has a narrow therapeutic index. Increased flecainide levels → QRS widening, PR prolongation, bradycardia, ventricular arrhythmias. Fluoxetine’s CYP2D6 inhibition can push flecainide into toxic range.
Anxiolytics [14]	Benzodiazepines	Generally safe	Minimal cardiac risk, even in patients with past myocardial infarctions; caution in severe conduction disease
Antipsychotics [18]	Thioridazine, chlorpromazine, haloperidol	Yes	QT prolongation, bradyarrhythmia, conduction block
	Risperidone, sertindole	Yes (high risk)	QT prolongation, bradycardia
Mood stabilizers [18]	Lithium	Yes	Sinus node dysfunction, bradycardia, AV block
	Carbamazepine	Yes	Sinus node dysfunction, conduction block
	Sodium valproate	Generally safe	Minimal cardiac risk
Stimulants [19]	Amphetamines, methylphenidate	Yes	Tachyarrhythmia, increased risk of SVT

Limitations

This report is subject to several limitations. As a single-patient case study, the findings cannot be generalized to all patients with SSS or other cardiac conduction disorders. The association between psychotropic agent use and the cardiac concerns suggests a medication-related effect; however, a direct causal relationship cannot be definitively established because multiple clinical factors, including the patient’s underlying arrhythmia, comorbid conditions, and concurrent medications, may have contributed to the observed outcomes. Additionally, serial serum flecainide concentrations were not obtained, limiting the ability to objectively confirm the extent of any pharmacokinetic interaction resulting from CYP2D6 inhibition by bupropion. Long-term follow-up data were also unavailable, preventing the assessment of the sustained safety and efficacy of alternative psychiatric treatment strategies. Furthermore, evidence regarding the use of psychotropic medications specifically in patients with SSS remains limited, and recommendations favoring agents such as sertraline and mirtazapine are largely extrapolated from broader cardiovascular populations rather than SSS-specific studies. Prospective investigations with larger cohorts are needed to better characterize psychotropic-associated arrhythmia risks, drug-drug interactions, and optimal treatment approaches in patients with conduction system disease.

## Conclusions

This case highlights the challenges of treating anxiety and insomnia in patients with SSS and SVT, where psychotropic medications may worsen conduction abnormalities or interact with antiarrhythmic therapy. Based on this case and the literature, non-pharmacological options, including cognitive behavioral therapy for insomnia, and pharmacological options, including sertraline and mirtazapine, were prioritized for their lower risk of conduction impairment and more favorable cardiovascular safety profiles. Medication selection requires careful attention to QT prolongation, metabolic pathways, and the cumulative burden of arrhythmia risk factors. In this patient, commonly used agents such as hydroxyzine, quetiapine, and bupropion introduced concerns for pro-arrhythmic potential or drug interaction with flecainide. ECG monitoring was emphasized to ensure early detection of adverse cardiac effects during treatment transitions. The case highlights the importance of coordinated management between psychiatry and cardiology to minimize risk and maintain symptom control. Broader clinical awareness of psychotropic cardiac risks is essential to guide safe prescribing in patients with conduction disorders. Further research is needed to strengthen evidence-based recommendations for psychiatric treatment in this population.
